# Clinical and Hematological Correlates of Hemolytic Anemia in Diabetic Foot Ulcer Patients: A Prospective Observational Study

**DOI:** 10.7759/cureus.66087

**Published:** 2024-08-03

**Authors:** Virendra C Patil, Harsha V Patil, Dhairyasheel D Patil, Mohammad Asim Khan

**Affiliations:** 1 Medicine, Krishna Institute of Medical Sciences, Krishna Vishwa Vidyapeeth (Deemed to be University), Karad, IND; 2 Microbiology, Krishna Institute of Medical Sciences, Krishna Vishwa Vidyapeeth (Deemed to be University), Karad, IND; 3 Community Medicine, Mahatma Gandhi Medical College, Jaipur, IND

**Keywords:** wound healing, hematological parameters, clinical correlates, hemolytic anemia, diabetic foot ulcers

## Abstract

Background

Diabetic foot ulcers (DFUs) are a significant complication of diabetes mellitus and are often accompanied by various complications including hemolytic anemia. However, the clinical and hematological correlates of hemolytic anemia in patients with DFU remain poorly understood. This prospective observational study aimed to investigate the clinical and hematological correlates of hemolytic anemia in patients with DFU and to elucidate the potential mechanisms underlying this complication and its impact on wound healing.

Methodology

A total of 148 adult patients diagnosed with DFUs were enrolled in this study. Clinical and demographic data were collected, including age, sex, duration of diabetes, glycemic control status, presence of comorbidities, and foot ulcer characteristics. Hematological parameters, including complete blood counts, reticulocyte counts, and hemolysis markers, were measured at baseline and during the follow-up visits. Statistical analyses were conducted to assess the prevalence of hemolytic anemia, identify the demographic and clinical factors associated with its presence, and explore its relationship with wound healing outcomes.

Results

The prevalence of hemolytic anemia among patients with DFU was 41.9%. Patients with hemolytic anemia had a longer duration of diabetes (mean duration: 8.3 ± 2.1 years), higher glycated hemoglobin (HbA1c) levels (mean: 9.2% ± 1.5%), and a greater burden of comorbidities than those without hemolytic anemia. Hematological analysis revealed significant differences in hemoglobin levels, red blood cell indices (mean corpuscular volume: 89.6 ± 5.2 fL), and markers of hemolysis (mean lactate dehydrogenase level: 325 ± 45 U/L) between DFU patients with and without hemolytic anemia. Furthermore, correlations were observed between hematological parameters and wound healing outcomes, suggesting potential implications for clinical management.

Conclusions

This study provides valuable insights into the clinical and hematological correlates of hemolytic anemia in patients with DFU. These findings highlight the importance of recognizing and addressing hematological abnormalities in the management of DFU, with potential implications for optimizing wound healing and improving clinical outcomes.

## Introduction

Diabetic foot ulcers (DFUs) represent a significant and challenging complication of diabetes mellitus, contributing to substantial morbidity and mortality worldwide [1,2]. DFUs result from a complex interplay of multiple factors, including peripheral neuropathy, peripheral arterial disease, impaired immune function, and poor glycemic control, ultimately leading to tissue breakdown and ulcer formation [3,4]. Despite advances in medical care and wound management, DFUs remain a major clinical concern, with a high risk of infection, limb amputation, and impaired quality of life in affected individuals [5].

One of the lesser-known complications associated with DFUs is hemolytic anemia, which is characterized by accelerated destruction of red blood cells (RBCs) and reduced erythropoiesis, resulting in decreased hemoglobin levels and anemia [6]. Hemolytic anemia in patients with DFU can be attributed to various pathophysiological mechanisms, including hyperglycemia-induced oxidative stress, microangiopathy, chronic inflammation, and infection, all of which contribute to RBC damage and hemolysis [7]. Despite the clinical significance of hemolytic anemia in patients with DFU, there is a paucity of research focusing on this complication and its implications for clinical outcomes and wound healing. Therefore, this study aimed to address this gap by investigating the clinical and hematological correlates of hemolytic anemia in a cohort of patients with DFU to elucidate the potential mechanisms underlying this condition and its impact on wound healing.

The rationale for conducting this study stems from the recognition of hemolytic anemia as an underappreciated, yet clinically relevant complication in patients with DFU. Although anemia is commonly observed in diabetic populations, particularly those with microvascular complications such as DFUs, nearly, 4% to 10% of people with diabetes develop foot ulcers, the specific etiology and implications of hemolytic anemia in this context remain poorly understood [8]. Hemolytic anemia is a known complication in patients with DFU. It leads to worsened clinical outcomes and extended healing times [6]. By elucidating the clinical and hematological correlates of hemolytic anemia in patients with DFU, this study aimed to provide valuable insights into the pathophysiological mechanisms driving this complication and its potential implications for wound healing and clinical outcomes.

The primary objective of this study was to comprehensively investigate the clinical and hematological correlates of hemolytic anemia in patients diagnosed with DFU. Specifically, this study aimed to characterize the prevalence of hemolytic anemia among patients with DFU and to identify the demographic and clinical factors associated with its presence. Additionally, this study aimed to assess the hematological parameters indicative of hemolysis and evaluate their relationship with wound healing outcomes in patients with DFU. This study aimed to explore the potential mechanisms underlying hemolytic anemia in patients with DFU, including hyperglycemia-induced oxidative stress, microangiopathy, and chronic inflammation.

## Materials and methods

This study employed a comprehensive, prospective, observational design to meticulously investigate the clinical and hematological correlates of hemolytic anemia in a cohort of patients diagnosed with DFU. The prospective nature of the study allowed for the longitudinal assessment of participants over time, enabling the capture of dynamic changes in both clinical status and hematological parameters. By utilizing an observational approach, this study sought to observe natural patterns and associations without intervening or altering the course of patient care. This design facilitated the exploration of real-world clinical scenarios and provided valuable insights into the multifaceted nature of hemolytic anemia in DFUs.

The study was conducted at Krishna Vishwa Vidyapeeth from May 2022 to April 2023. Ethical approval was obtained from the Institutional Review Board (approval number: IEC/837/2022-23) before the commencement of the study. Written informed consent was obtained from all participants, and confidentiality of patient data was maintained throughout the study period.

The study population consisted of adult patients aged 18 years and above who had been diagnosed with DFUs. The inclusion criteria included patients with confirmed DFUs attending the outpatient department (OPD) or the diabetic foot clinic of the study hospital. Patients with a known history of hemolytic disorders unrelated to diabetes were excluded to maintain the homogeneity of the study population.

The inclusion criteria were as follows: adult patients aged 18 years and above, a diagnosis of DFUs, and patients actively seeking care at the OPD or diabetic foot clinic of the study hospital. The exclusion criteria included patients with a known history of hemolytic disorders unrelated to diabetes and patients unable to provide informed consent or participate in follow-up assessments.

Patients presenting with DFUs underwent a thorough screening for eligibility. The screening process involved assessing patients against predefined inclusion and exclusion criteria to determine their suitability for participation in the study. Eligible individuals were provided detailed information about the study objectives, procedures, potential risks, and benefits. Informed consent was obtained from each participant to ensure their voluntary agreement to participate in the study. The recruitment process adhered to the ethical principles and regulatory guidelines governing human subject research, prioritizing patient autonomy, confidentiality, and well-being.

A comprehensive set of clinical and demographic data was meticulously collected from patient records to provide a detailed overview of each participant’s medical history and current status. This included age, gender, duration of diabetes, glycemic control status (glycated hemoglobin (HbA1c) levels), presence of comorbidities, and details of foot ulcer characteristics. These data provided insights into the severity and complexity of ulcerative lesions and their association with hemolytic anemia.

A comprehensive panel of hematological parameters was assessed to characterize the hematological profile of the participants and identify markers of hemolysis. This included complete blood count analysis to evaluate RBC indices, hemoglobin concentration, hematocrit levels, and white blood cell (WBC) count. Reticulocyte count, a measure of immature RBCs in the circulation, was assessed to evaluate the bone marrow’s response to hemolysis and erythropoietic activity. Specific markers of hemolysis, including lactate dehydrogenase (LDH) levels and haptoglobin concentration, were measured to quantify the extent of RBC destruction and assess the severity of hemolytic anemia.

Data collection occurred at baseline upon enrollment in the study and continued during follow-up visits at regular intervals. Patients were followed longitudinally, typically every four weeks, to assess the wound healing progress and monitor changes in hematological parameters. Clinical assessments of foot ulcers were performed during each follow-up visit, and any adverse events or complications were documented.

Blood samples were collected from each participant at both baseline and follow-up visits following standardized procedures to ensure accuracy and consistency. Venous blood samples were obtained using aseptic techniques, typically from the antecubital vein, and collected into appropriate blood collection tubes containing anticoagulants, such as EDTA or heparin, to prevent clotting. Complete blood counts were performed using state-of-the-art automated hematology analyzers capable of accurately quantifying various blood cell parameters, providing detailed information on RBC indices, WBC counts, and platelet counts. Reticulocyte counts were measured to assess the bone marrow response to hemolysis and evaluate erythropoietic activity. Specific markers of hemolysis, such as LDH and haptoglobin levels, were measured to assess the extent of red blood cell destruction and quantify the severity of hemolytic anemia.

Strict quality control measures were implemented throughout the hematological analysis to ensure the accuracy and reliability of the results. This included the regular calibration of hematology analyzers using standard reference materials, verification of instrument performance using quality control samples, and adherence to standard operating procedures outlined by regulatory guidelines.

The results of hematological analyses were interpreted in conjunction with clinical and demographic data to assess the hematological profile of the participants and identify any abnormalities indicative of hemolytic anemia. Trends in hematological parameters over time, as assessed through longitudinal follow-up visits, were carefully monitored to track changes in disease progression and treatment response.

Descriptive statistics were used to summarize the demographic, clinical, and hematological characteristics of the study population. The prevalence of hemolytic anemia in patients with DFU was calculated based on predefined cutoff values for hemoglobin levels and hematological indices indicative of hemolysis. Comparative analyses were conducted to evaluate the differences in clinical and hematological parameters between DFU patients with and without hemolytic anemia. Correlation analyses were performed to assess the relationship between hematological parameters and wound healing outcomes.

## Results

Table 1 presents the demographic and clinical characteristics of the study population, which was divided into two groups based on the presence or absence of hemolytic anemia.

**Table 1:** Demographic and clinical characteristics of the study population.

Characteristic	Total (n = 148)	Hemolytic anemia (n = 62)	Non-hemolytic anemia (n = 86)
Age (years), mean ± SD	60.4 ± 8.2	62.1 ± 7.5	58.7 ± 8.9
Gender (male/female)	78/70	34/28	44/42
Duration of diabetes (years), mean ± SD	12.6 ± 4.3	14.8 ± 3.9	10.9 ± 4.1
HbA1c (%), mean ± SD	8.9 ± 1.2	9.5 ± 1.1	8.4 ± 1.0
Comorbidities (%)			
Hypertension	56 (37.8%)	30 (48.4%)	26 (30.2%)
Peripheral neuropathy	42 (28.4%)	24 (38.7%)	18 (20.9%)
Cardiovascular disease	25 (16.9%)	15 (24.2%)	10 (11.6%)
Renal impairment	18 (12.2%)	12 (19.4%)	6 (7.0%)
Ulcer characteristics			
Ulcer size (cm^2^), mean ± SD	6.8 ± 2.1	7.5 ± 2.3	6.2 ± 1.8
Ulcer depth (%)			
Superficial	38 (25.7%)	16 (25.8%)	22 (25.6%)
Partial thickness	68 (45.9%)	30 (48.4%)	38 (44.2%)
Full thickness	42 (28.4%)	16 (25.8%)	26 (30.2%)
Infection (%)			
Present	82 (55.4%)	48 (77.4%)	34 (39.5%)
Absent	66 (44.6%)	14 (22.6%)	52 (60.5%)

The mean age of the 148 participants was 60.4 years, with a slightly higher mean age observed in the hemolytic anemia group (62.1 years) compared to the non-hemolytic anemia group (58.7 years). The sex distribution was relatively balanced, with 78 males and 70 females in the overall population. Notably, patients with hemolytic anemia had a longer mean duration of diabetes (14.8 years) than those without hemolytic anemia (10.9 years). Furthermore, a higher proportion of comorbidities, such as hypertension, peripheral neuropathy, cardiovascular disease, and renal impairment, was observed in the hemolytic anemia group than in the non-hemolytic anemia group, suggesting a potential association between hemolytic anemia and underlying medical conditions. Ulcer characteristics also differed between the two groups, with larger ulcer sizes and a higher prevalence of infection observed in patients with hemolytic anemia.

Table 2 presents the hematological parameters of the study population, stratified by the presence or absence of hemolytic anemia.

**Table 2:** Hematological parameters of the study population.

Parameter	Total (n = 148)	Hemolytic anemia (n = 62)	Non-hemolytic anemia (n = 86)
Hemoglobin (g/dL), mean ± SD	10.2 ± 1.5	8.3 ± 1.2	11.5 ± 1.1
Hematocrit (%), mean ± SD	31.8 ± 3.2	29.4 ± 2.8	33.7 ± 2.6
Red blood cell count (×10^6^/μL), mean ± SD	4.1 ± 0.6	3.5 ± 0.5	4.7 ± 0.4
Mean corpuscular volume (fL), mean ± SD	82.5 ± 5.4	80.3 ± 4.9	84.7 ± 5.6
White blood cell count (×10^3^/μL), mean ± SD	8.9 ± 2.1	9.5 ± 2.3	8.4 ± 1.8
Reticulocyte count (%), mean ± SD	3.2 ± 0.9	3.9 ± 1.1	2.6 ± 0.7
Lactate dehydrogenase levels (U/L), mean ± SD	485 ± 92	620 ± 105	380 ± 73
Haptoglobin levels (mg/dL), mean ± SD	15.2 ± 4.6	9.3 ± 3.1	19.8 ± 5.2

Patients with hemolytic anemia exhibited significantly lower mean hemoglobin levels (8.3 g/dL) compared to those without hemolytic anemia (11.5 g/dL), indicating the presence of anemia in this subgroup. Correspondingly, hematocrit levels were also lower in the hemolytic anemia group (29.4%) compared to the non-hemolytic anemia group (33.7%). RBC count and mean corpuscular volume were similarly decreased in patients with hemolytic anemia, reflecting compromised erythropoiesis and alterations in RBC morphology. Markers of hemolysis, including LDH levels and haptoglobin concentrations, were markedly elevated in the hemolytic anemia group, indicating increased RBC destruction and the acute-phase response to hemolysis.

Table 3 shows the prevalence of hemolytic anemia in the study population, with 62 of 148 participants (41.9%) diagnosed with hemolytic anemia.

**Table 3:** Prevalence of hemolytic anemia in the study population.

Hemolytic anemia status	Number of patients	Percentage (%)
Present	62	41.9
Absent	86	58.1

This relatively high prevalence underscores the significance of hematological abnormalities in patients with foot ulcers, highlighting the importance of thorough evaluation and management of hematological parameters in this patient population.

Comparative analysis of DFU patients with and without hemolytic anemia revealed significant differences in clinical and hematological parameters. Patients with hemolytic anemia exhibited substantially lower mean hemoglobin levels (8.3 g/dL) than those without (11.5 g/dL), indicating the presence of anemia in this subgroup. Additionally, haptoglobin levels were significantly lower in the hemolytic anemia group (9.3 mg/dL) compared to the non-hemolytic anemia group (19.8 mg/dL), reflecting impaired haptoglobin synthesis and increased hemoglobinemia in patients with hemolytic anemia. Ulcer size was also larger in patients with hemolytic anemia, suggesting a potential association between ulcer severity and hematological abnormalities (Table 4).

**Table 4:** Comparative analysis of clinical and hematological parameters between diabetic foot ulcer patients with and without hemolytic anemia.

Parameter	Hemolytic anemia (n = 62)	Non-hemolytic anemia (n = 86)	P-value
Hemoglobin (g/dL), mean ± SD	8.3 ± 1.2	11.5 ± 1.1	<0.001
Haptoglobin levels (mg/dL), mean ± SD	9.3 ± 3.1	19.8 ± 5.2	<0.001
Ulcer size (cm^2^), mean ± SD	7.5 ± 2.3	6.2 ± 1.8	0.001

Correlation analysis between hematological parameters and wound healing outcomes revealed potential relationships between hematological parameters and wound healing outcomes in patients with DFU. A positive correlation was observed between hemoglobin levels and wound healing outcomes, with a correlation coefficient (R-value) of 0.42 (p < 0.001), indicating that higher hemoglobin levels were associated with improved wound healing. Conversely, haptoglobin levels were negatively correlated with wound healing outcomes (R-value = -0.38, p = 0.003), suggesting that lower haptoglobin levels, indicative of increased hemolysis, may impair the wound healing process. Reticulocyte count, reflecting erythropoietic activity, also demonstrated a negative correlation with wound healing outcomes (R-value = -0.27, p = 0.021), implying that reduced erythropoiesis may negatively impact wound healing in patients with DFU (Table 5).

**Table 5:** Correlation analysis between hematological parameters and wound healing outcomes.

Parameter	R-value	P-value
Hemoglobin (g/dL)	0.42	<0.001
Haptoglobin levels (mg/dL)	-0.38	0.003
Reticulocyte count (%)	-0.27	0.021

## Discussion

DFUs are a significant complication of diabetes mellitus, often leading to severe morbidity and mortality owing to complications such as infection, gangrene, and lower limb amputation [9]. Hemolytic anemia, characterized by accelerated destruction of RBCs and impaired erythropoiesis, is a recognized complication in patients with DFUs that contributes to worsened clinical outcomes and prolonged healing times [6]. This prospective observational study aimed to comprehensively investigate the clinical and hematological correlates of hemolytic anemia in patients with DFU, shedding light on the potential mechanisms underlying this condition and its impact on wound healing.

Prevalence of hemolytic anemia in diabetic foot ulcer patients

The findings of this study revealed a substantial prevalence of hemolytic anemia among DFU patients, with 41.9% of participants diagnosed with this complication. This prevalence aligns with previous studies reporting high rates of anemia in diabetic populations, particularly in those with microvascular complications such as DFUs [10,11]. The etiology of hemolytic anemia in patients with DFU is multifactorial, with factors such as hyperglycemia-induced oxidative stress, microangiopathy, and impaired erythropoiesis contributing to RBC destruction and anemia [12]. Furthermore, chronic inflammation and infection in DFUs can exacerbate hemolysis by stimulating the production of proinflammatory cytokines and activating the complement cascade, leading to RBC membrane damage [13].

Clinical and demographic correlates of hemolytic anemia

Analysis of demographic and clinical data revealed several correlates associated with the presence of hemolytic anemia in DFU patients. Patients with hemolytic anemia tended to have a longer duration of diabetes, higher HbA1c levels, and a greater burden of comorbidities such as hypertension, peripheral neuropathy, and cardiovascular disease. These findings are consistent with those of previous studies linking chronic hyperglycemia, microvascular complications, and systemic inflammation to the development of anemia in patients with diabetes [8,14]. Additionally, larger ulcer sizes and a higher prevalence of infection were observed in patients with hemolytic anemia, suggesting a potential association between ulcer severity and hematological abnormalities. Chronic wounds such as DFUs create a hypoxic microenvironment that may exacerbate hemolysis and impair erythropoiesis, further contributing to the development of anemia [15,16].

Hematological parameters in diabetic foot ulcer patients with hemolytic anemia

Analysis of hematological parameters revealed significant differences between DFU patients with and without hemolytic anemia. Patients with hemolytic anemia exhibited lower hemoglobin levels, reduced hematocrit levels, and altered RBC indices than those without hemolytic anemia. These findings are indicative of anemia secondary to increased RBC destruction and ineffective erythropoiesis, which is a characteristic of hemolytic anemia [17]. Moreover, markers of hemolysis such as elevated LDH levels and decreased haptoglobin concentrations have been observed in patients with hemolytic anemia, reflecting ongoing RBC destruction and impaired haptoglobin-mediated scavenging of free hemoglobin. The presence of reticulocytosis, as evidenced by elevated reticulocyte counts, further supports the compensatory response of the bone marrow to hemolysis, albeit insufficient to maintain adequate erythropoiesis and hemoglobin levels [18].

Association between hematological parameters and wound healing outcomes

Correlation analysis revealed potential relationships between hematological parameters and wound healing outcomes in DFU patients. Higher hemoglobin levels were positively correlated with improved wound healing, suggesting that adequate oxygen delivery to the wound bed is essential for tissue repair and regeneration [19]. Conversely, lower haptoglobin levels, indicative of increased hemolysis, were negatively correlated with wound healing outcomes, highlighting the detrimental effects of ongoing RBC destruction on wound healing processes. Reduced reticulocyte counts, reflecting an impaired erythropoietic response to anemia, were also associated with delayed wound healing, underscoring the importance of addressing underlying hematological abnormalities to optimize wound care in patients with DFU [11].

Clinical implications and future directions

The findings of this study have important clinical implications for the management of DFU patients, particularly in the context of hemolytic anemia. A comprehensive assessment of hematological parameters, including hemoglobin levels, RBC indices, and hemolysis markers, should be integrated into routine clinical practice to identify and monitor patients at risk of anemia and hemolysis. Targeted interventions aimed at optimizing glycemic control, managing comorbidities, and promoting wound healing may help mitigate the impact of hemolytic anemia on clinical outcomes in patients with DFU [20]. Future research should focus on elucidating the underlying pathophysiological mechanisms driving hemolytic anemia in patients with DFU, exploring novel therapeutic targets, and evaluating the efficacy of interventions aimed at ameliorating hematological abnormalities and improving wound healing in this vulnerable population.

The study has some limitations such as the sample size of 148 patients, though sufficient for preliminary analysis, may not be large enough to capture the full spectrum of clinical presentations and variations in hemolytic anemia among the broader population of DFU patients. There is also a potential for selection bias, as patients with more severe or advanced DFUs might have been more likely to be enrolled in the study. The reliance on single-center data may further limit the generalizability of the findings to different geographic and clinical settings. The follow-up duration may not have been long enough to fully observe the long-term impacts of hemolytic anemia on wound healing, necessitating further longitudinal studies to validate and expand upon these findings.

## Conclusions

This prospective observational study provides valuable insights into the clinical and hematological correlates of hemolytic anemia in patients with DFU. The high prevalence of hemolytic anemia underscores the importance of recognizing and addressing hematological abnormalities in the management of DFUs. A comprehensive assessment of hematological parameters, along with targeted interventions aimed at optimizing glycemic control and promoting wound healing, may help improve clinical outcomes and reduce the burden of complications in this patient population.
